# A Statement to American Dentists Practicing in Europe

**Published:** 1899-05

**Authors:** 


### A Statement

### TO AMERICAN DENTISTS PRACTICING IN EUROPE.

By the Foreign Relations Committee of the N. A. of D. F. of America.

At a special meeting of the Foreign Relations Committee of the National Association of Dental Faculties of America, held in Cincinnati, Ohio, December 27, 28 and 29, 1898, in accordance with the instructions given at the annual meeting of the Association, held in Omaha, Neb., August, 1898, certain of their confrerers living in Europe were appointed to form the nucleus of an advisory body, the membership of which it is their purpose to increase to the number of three for each of the principal countries of Europe, as soon as they shall become thoroughly convinced as to the best manner of organizing such Board, and fully informed concerning nominations for membership therein.

The Foreign Relations Committee feels itself restricted in its action by the instructions given it by the National Association, and cannot at present clearly see its way to do more than to lay the foundation for future more comprehensive action. It believes that any precipitancy on its part, in the absence of a full and clear comprehension of the exact status of American dentistry in Europe, might do great injury to the cause of American dental education, and prejudice us greatly in the eyes of foreign professional men. The members also realize that until there is a better understanding of professional affairs in Europe by Americans in this country, it would be easy to injure our colleges by creating a prejudice that would be baseless and unjust. No possible harm can result from the exercise of great care, and even from delay on our part in the completion of the appointment to this Board, while radical action in the absence of definite knowledge, would be certain to work evil.

Hence the Committee has not felt itself justified in doing more at present than to make a few appointments that are entirely unopposed, and to go no further than to commit to such members of the Foreign Board the responsibility of examining the credentials of students making application from foreign countries for matriculation in American dental colleges, the advising of the Foreign Relations Committee of the requirements demadned for practice in such countries, the number, names and professional status of the holders of American dental degrees abroad, and the giving of such other information as may prove of benefit to the National Association of Dental Faculties. For the information of such Foreign Board the Committee has unanimously adopted the following expression of opinion:

FIRST. "The proposed Board shall be known as the European Advisory Board of the Foreign Relations Committee of the National Association of Dental Faculties of America.

SECOND. "Its objects shall be to ascertain the standing and reputation of institutions in foreign countries giving instruction in dental subjects, the character of instruction imparted, the different courses of study, the length of term, the requirements for admission, and the form of certificate given entitling the holder to practice dentistry in such foreign countries.

THIRD. "To examine the certificates of Europeans who purpose coming to this country to complete their dental studies after a course more or less complete abroad, to report upon the value of such certificates, and how much credit should be allowed them in American dental colleges as a consequence, and to communicate to the Chairman of the Foreign Relations Committee any further facts that may serve as a guide to the deans of American dental colleges in the reception and proper assignment of such students.

FOURTH. "To furnish the Foreign Relations Committee with the names of such persons as may have come, or who may purpose coming, to the United States for professional instruction, and whom they may believe to be unworthy of reception in American colleges, with the facts upon which such belief is based.

FIFTH. "To obtain for the Foreign Relations Committee, as far as is practicable, a complete list of all American graduates practicing in Europe, giving names of the schools that issued their diplomas, together with date of graduation, and the general reputation and status of such graduates."

The Foreign Relations Committee desires explicitly to say that while it is not authorized to extend the scope of its present action, and deems it unwise on its part to go further in defining the duties of the European Advisory Board, it is heartily in sympathy with American dental graduates abroad in their efforts to obtain a due recognition of the American dental degree in Europe, and pledges itself, whenever it believes the time is ripe for definite action, to take any steps which in its opinion will tend to bring about so desirable an object.

The Committe desires to announce the following appointments to the European Advisory Board:

Great Britain, Dr. W. Mitchell, London; Holland and Belgium, Dr. J. E. Grevers, Amsterdam; Denmark, Norway and Sweden, Dr. Elof Forberg, Stockholm; Russia, —; Germany, —; Austria and Hungary, —; Italy and Greece, Dr. Albert T. Webb, Rome; France, Dr. J. H. Spaulding, Paris; Spain and Portugal, —; Switzerland and Turkey, Dr. L. C. Bryan, Basle.

*Committee.*

S. H. Guilford,

J. D. Patterson,

T. W. Brophy,

H. W. Morgan,

W. C. Barrett, Chairman,

208 Franklin St., Buffalo, N. Y., U. S. A.

